# Multiple Cases of Bacterial Sequence Erroneously Incorporated Into Publicly Available Chloroplast Genomes

**DOI:** 10.3389/fgene.2021.821715

**Published:** 2022-01-13

**Authors:** Aaron J. Robinson, Hajnalka E. Daligault, Julia M. Kelliher, Erick S. LeBrun, Patrick S. G. Chain

**Affiliations:** Los Alamos National Laboratory, Biosecurity and Public Health Group, Bioscience Division, Los Alamos, NM, United States

**Keywords:** chloroplast, plastome, sequence contamination, public sequence databases, genome repositories

## Abstract

Public sequencing databases are invaluable resources to biological researchers, but assessing data veracity as well as the curation and maintenance of such large collections of data can be challenging. Genomes of eukaryotic organelles, such as chloroplasts and other plastids, are particularly susceptible to assembly errors and misrepresentations in these databases due to their close evolutionary relationships with bacteria, which may co-occur within the same environment, as can be the case when sequencing plants. Here, based on sequence similarities with bacterial genomes, we identified several suspicious chloroplast assemblies present in the National Institutes of Health (NIH) Reference Sequence (RefSeq) collection. Investigations into these chloroplast assemblies reveal examples of erroneous integration of bacterial sequences into chloroplast ribosomal RNA (rRNA) loci, often within the rRNA genes, presumably due to the high similarity between plastid and bacterial rRNAs. The bacterial lineages identified within the examined chloroplasts as the most likely source of contamination are either known associates of plants, or co-occur in the same environmental niches as the examined plants. Modifications to the methods used to process untargeted ‘raw’ shotgun sequencing data from whole genome sequencing efforts, such as the identification and removal of bacterial reads prior to plastome assembly, could eliminate similar errors in the future.

## Introduction

Publicly available sequence databases, such as those in the International Nucleotide Sequence Database Collaboration (INSDC), are fundamental resources for many types of bioinformatic analyses. With the increased availability of sequencing and a wide array of methods designed for routine use by genomics and bioinformatic novices, there is a constant need to monitor sequence entries and try to assess the quality and veracity of data within these public resources. The errors present in curated and otherwise trusted databases (Steinegger and Salzberg, 2020; Orakov et al., 2021), such as the National Center for Biotechnology Information (NCBI) RefSeq, are of particular concern as both sequences and associated taxonomic designations reported in these databases are often blindly accepted as accurate by the majority of users, and this database is a common source of reference genomes used for comparative genomic analyses. Given that the nature of most bioinformatic analyses involve similarity searches to references in these databases, it is not uncommon to see transference of annotation and genome errors to other projects or analyses, making it imperative to quickly identify and correct any erroneous submissions within these trusted databases, prior to the propagation of errors.

The NCBI RefSeq database contains a large number of plant and algal nuclear and chloroplast genome reference assemblies, many of which are derived from taxa important to either environmental functioning, agriculture, or medicine. Unfortunately, organelle genome references are not curated as stringently as their nuclear counterparts, increasing the likelihood that erroneous sequences may be published. Additionally, nuclear genomes are almost always published along with the raw sequencing data used to generate the assembly, but based on our examinations, it appears to be quite uncommon to find links to raw data for organelle genomes found in RefSeq or GenBank databases. Particularly in the case of plastids, which are often examined independently from the nuclear genome, the absence of this supporting sequencing data makes it challenging to identify, assess, and correct errors in published assemblies. Additionally, previous screens of the RefSeq and GenBank databases to assess genome assembly quality in terms of potential contamination, to our knowledge, have not included plastome sequences. Evaluation of plastomes would also require special considerations, given their unique evolutionary relationships with bacteria, which complicate assessment of contamination.

While screening plastid sequences in the NCBI RefSeq plastid genome collection (https://www.ncbi.nlm.nih.gov/genome/organelle/), we identified several chloroplast genomes that contained sequence signatures more similar to bacteria than chloroplast. Herein, we present several problematic chloroplast references present in RefSeq which each contain variable and unique bacterial sequence contamination in the regions containing the 16S and 23S rRNA genes. Analysis of the raw sequencing data used to generate these assemblies indicates the inclusion of bacterial DNA. One possible source of the bacterial DNA detected in these chloroplast sequencing projects is potential bacterial associates of the host plant or its environment. This work highlights several concerns unique to the NCBI organelle RefSeq database, and suggests potential changes to help reduce the observed issues.

## Methods

All examined chloroplast genome assemblies were obtained from the 11/2020 NCBI RefSeq release of plastid sequences (https://ftp.ncbi.nlm.nih.gov/refseq/release/plastid/). The chloroplast assemblies examined in this work were identified as a result of mapping bacterial metagenomic reads to the entire NCBI RefSeq plastid genome collection (5,569 Plastomes total). Bacterial reads mapped to several chloroplast genome references at a relatively high frequency, which seemed abnormal and were thus investigated more closely. The read mapping results for each of these selected chloroplast assemblies all had similar mapping results, with bacterial reads piling up in the regions containing the 16S and 23S rRNA genes. Annotated chloroplast, plant nuclear, and bacterial sequences and genome assemblies were all obtained from either NCBI RefSeq or GenBank (accessions and sequence ranges provided in results). Alignments and comparisons to the NCBI nucleotide collection (nt) were performed using the BLASTN algorithm (https://blast.ncbi.nlm.nih.gov/Blast.cgi). Visual alignments presented in figures were generated using the mVISTA and AVID alignment program (Mayor et al., 2000; Frazer et al., 2004) with default settings. All read-based analyses were performed using the EDGE v2.4.0 bioinformatics platform (Li et al., 2017). The following quality-control parameters were used for all examined Illumina and IonTorrent datasets provided by the original submitters: bases from the ends of reads with a Phred score below 20 were trimmed, minimum read length after trimming had to be at least 50 bp, trimmed reads containing 10 or more continuous “N” bases were removed, and sequences comprised of 85% or more low complexity sequence (e.g., mono-/di-nucleotide sequence) were removed. Detailed information on these quality-control parameters can be found in the documentation for FaOCs (https://github.com/LANL-Bioinformatics/FaQCs). Taxonomic classification of quality-controlled reads was performed using GOTTCHA2 (Freitas et al., 2015) with a database generated from the NCBI bacterial RefSeq90 complete genome collection, which contained 14,529 bacterial genomes. Additionally, all reads were aligned to the bacterial RefSeq collection using the BWA-MEM algorithm (Li, 2013). Reference based analysis and read mapping were also performed using the BWA-MEM algorithm. Bacterial reads were filtered from Illumina and IonTorrent sequencing datasets by aligning reads to both the NCBI bacterial RefSeq collection and/or selected bacterial references, and any sequences with 90% or greater sequence identity were removed from the dataset. Alignments for phylogenetic analysis were performed using Clustal Omega v1.2.4 (Sievers et al., 2011) and phylogenetic trees were produced using RAxML v8.2.12 (Stamatakis, 2014) using the rapid bootstrap algorithm with 100 iterations and the GTRCAT model of substitution. Phylogenetic trees were edited using FigTree v1.4.4 (https://github.com/rambaut/figtree), and only bootstrap values ≥75 are shown. The sequence data used to generate the erroneous *P. japonica* (NC_037440.1), *R. parvula* (NC_031180.2) and *D. unilobum* (NC_035853.1) chloroplast references have been uploaded to the NCBI SRA database (Supplementary Table S1).

## Results

The rRNA regions from several RefSeq chloroplast genome assemblies were examined for potential bacterial sequence contamination. These chloroplast genomes, which represent a species of orchid and two red algal species, were all obtained and submitted by separate research groups, using distinct methods for assembly and annotation (Supplementary Table S1). When possible, the raw sequencing data used to generate these chloroplast genome assemblies was screened for bacterial contamination, and bacterial filtered data was compared to the assembly to assess impacts. The ribosomal RNA (rRNA) regions in the original assemblies appear to contain sequences derived from bacterial reads, ranging from relatively short stretches of sequence to an extreme case where ∼1.5 kb of bacterial-like 16S sequence was inserted immediately adjacent and upstream of the annotated chloroplast 16S sequence. Descriptions of these anomalous findings detected within the published and annotated rRNA sequences from these chloroplast genome assemblies are detailed below.

### Two *Platanthera japonica* Chloroplast Assemblies Contain Bacterial 23S rRNA Sequence

**FIGURE 1:**
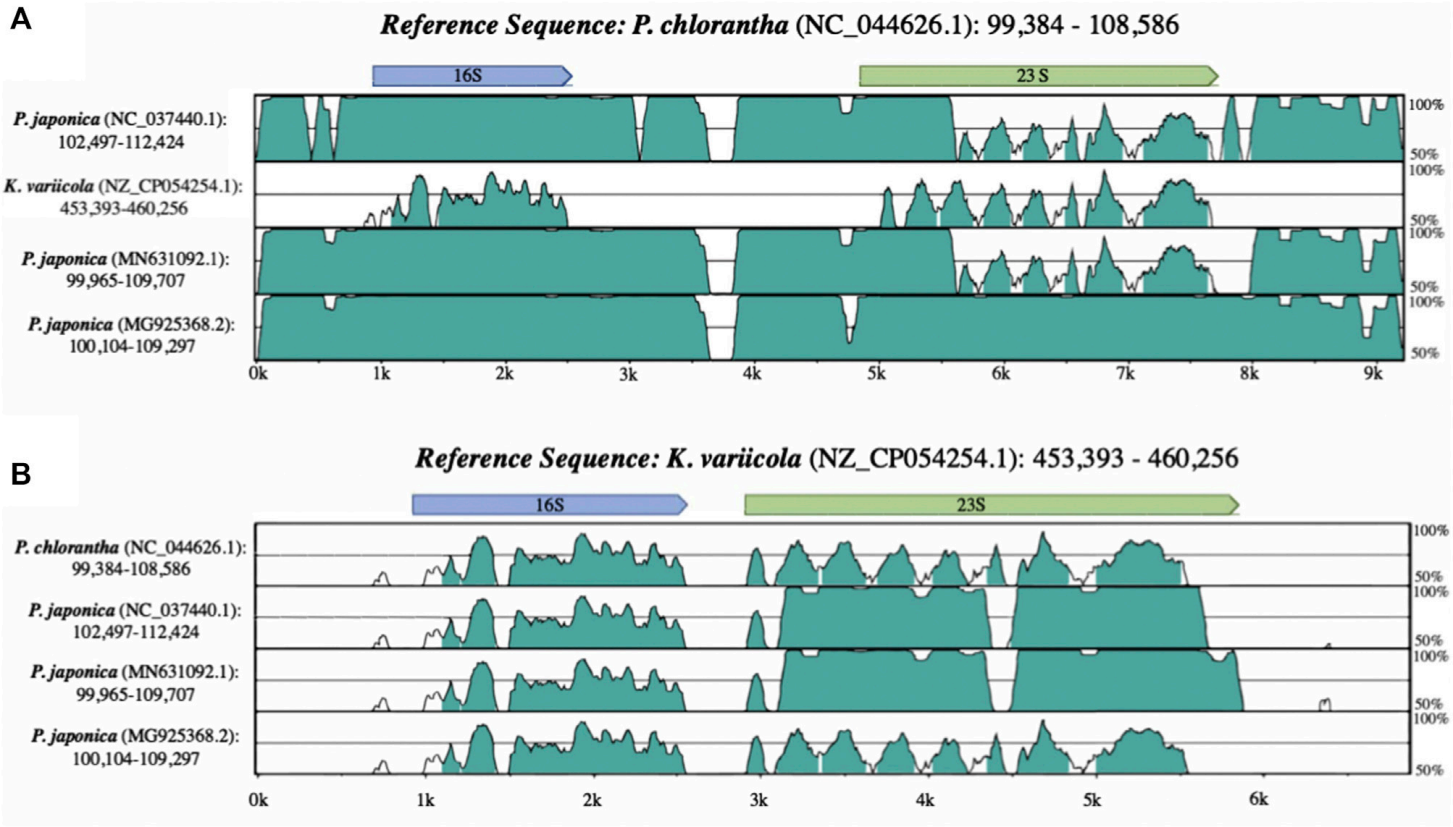
Alignments showing similarity between *Platanthera* chloroplast and *Klebsiella variicola* rRNA sequences when **(A)** *P. chlorantha* (NC_044626.1) and **(B)** *K. variicola* (NZ_CP054254.1) are used as references.

Alignments between the annotated rRNA operon sequences from the *Platanthera japonica* chloroplast (NCBI accession: NC_037440.1) and *Platanthera chlorantha* (NC_044626.1) show that while the majority of the region is highly similar between both *Platanthera* species, parts of the 23S gene are highly dissimilar (Figure 1). The annotated *P. japonica* 23S sequence was then aligned to the NCBI nucleotide collection (nt) using BLASTN, which revealed the sequence was highly similar to sequences from the bacterial genus *Klebsiella* (>99%). Taxonomic classification of the reads used to generate the *P. japonica* chloroplast assembly (NC_037440.1) indicated the presence of contaminating *Klebsiella* reads in the dataset (Supplementary Table S1). Alignments presented in Figure 1 demonstrate that the 23S sequences from *Klebsiella variicola* strain FH-1 (NZ_CP054254.1) and the *P. japonica* chloroplast (NC_037440.1) are more similar to each other than either is to the *P. chlorantha* (NC_044626.1) sequence. Once bacterial reads were removed from the dataset, the filtered reads were mapped to *P. chlorantha* (NC_044626.1) chloroplast assembly to obtain the correct *P. japonica* (NC_037440.1) 23S sequence. This corrected sequence was provided to the original submitters of the chloroplast genome assembly and was compared to the updated assembly (generated by original submitters) to ensure proper representation (Supplementary Table S1).

A separate *P*. *japonica* chloroplast assembly (MN631092) was subsequently sequenced and published after the release of the erroneous *P. japonica* (NC_037440.1) plastome sequence. Both plastomes were derived from leaves collected from the same geographic location, but different references were utilized by the two groups to guide the assembly (Supplementary Table S1) (Dong et al., 2018; Zhang et al., 2020). BLASTN alignments between these two *P*. *japonica* chloroplast assemblies revealed a high overall similarity (95% coverage, 99.45% identity), including identical 23S sequences in both chloroplast assemblies. Therefore, two separate erroneous chloroplast sequences exist within GenBank for this plant species and after sharing our findings with the original submitters and NCBI staff, both the GenBank (MG925368.2) and RefSeq (NC_037440.2) chloroplast entries have now been updated with the corrected 23S sequences (Supplementary Table S1).

### Bacterial Sequences Present in *Rhodochaete parvula* rRNA Regions

**FIGURE 2:**
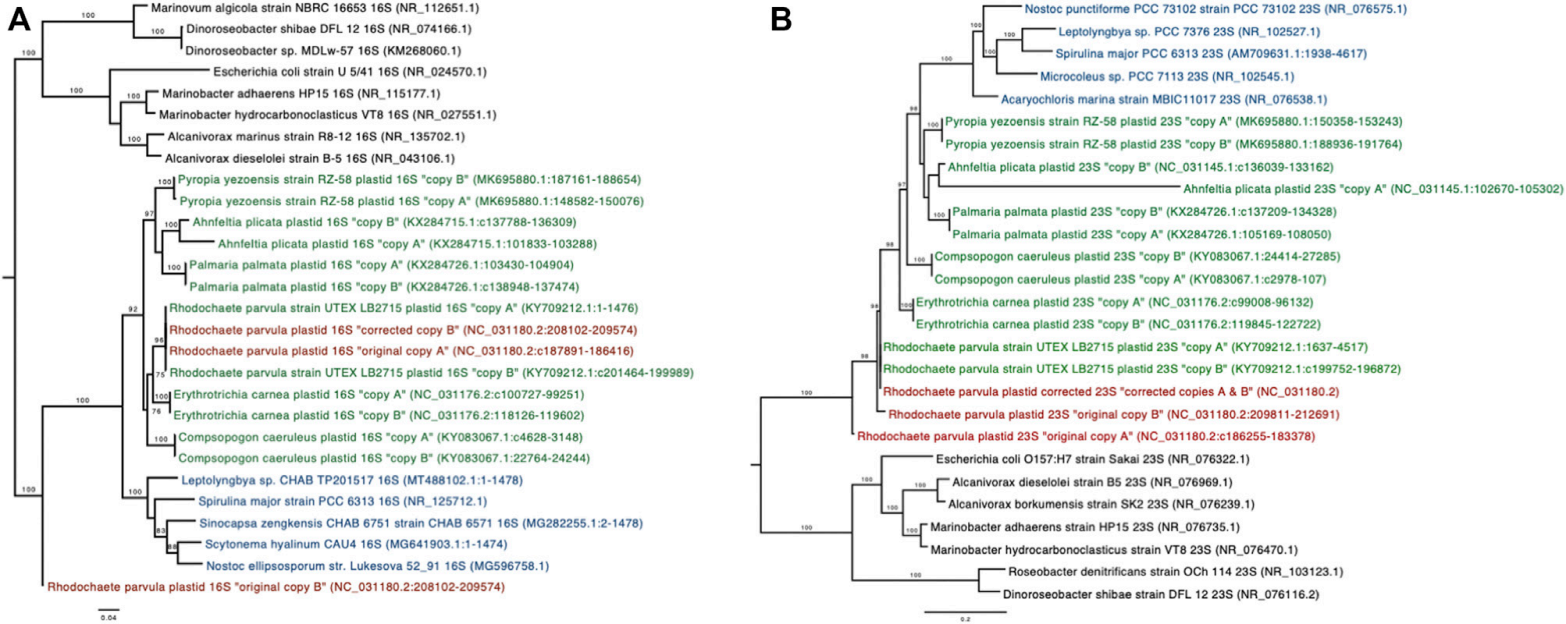
Phylogenetic trees of the 16S **(A)** and 23S **(B)** sequences, showing relationships between bacterial (black), cyanobacterial (blue), chloroplast sequences (green) and the *R. parvula* sequences (red). The original *R. parvula* chloroplast 16S and 23S sequences (2 copies each) from RefSeq assembly (NC_031180.2) are included as well as the corrected sequences obtained after removal of bacterial reads from the original sequencing dataset. NCBI accession identifiers and sequence ranges are shown in parentheses. Branches with bootstrap support greater than 75% (100 bootstrap replicates) are shown.

Alignments of the annotated 16S sequences from the *Rhodochaete parvula* (NC_031180.2) chloroplast assembly to the NCBI nucleotide collection (nt) revealed that one of the sequences (“copy A”) was more similar (90–95% identity) to other chloroplast sequences, while the other sequence (“copy B”) shared only 86.64% identity with the top chloroplast match in the database. Phylogenetic trees produced from an alignment of 16S sequences from this *R. parvula* assembly, closely related chloroplasts, cyanobacteria, and bacteria indicate that the *R. parvula* 16S copy A (NC_031180.2:c187891-186416) sequence is most closely related to sequences from another *R. parvula* isolate (KY709212.1), while copy B (NC_031180.2:208102-209574) is more distantly related to all examined plastid and cyanobacterial sequences (Figure 2A). Similar phylogenetic analyses conducted with the 23S sequences indicate more distant relationships between both copies of *R. parvula* sequences and sequences from the same plant species than what is observed in other plant species (Figure 2B). Closer examination of these sequences revealed that short stretches in copy B of the 16S gene and in both 23S copies were highly similar to bacterial rRNA sequences from the genus *Marinobacter.* This led to the hypothesis that bacterial sequences were erroneously incorporated into this chloroplast assembly. Read-based taxonomic classification of the raw sequencing data used to generate this chloroplast assembly (SRA accession SRR16979013) indicated that 33% of the total reads were classified as bacterial using the GOTTCHA2 software (see Methods; Supplementary Table S1). Corrected versions of the 16S and 23S sequences from this *R. parvula* (NC_031180.2) assembly were generated by filtering bacterial reads prior to reassembly (see Methods), and these corrected sequences were confirmed to be highly similar to sequences from the other examined *R. parvula* isolate (KY709212.1) (Figure 2). These corrected sequences were provided to the original submitters of the chloroplast genome assembly and were compared to the updated assembly (generated by original submitters) to ensure proper representation. After sharing our findings with the original submitters and NCBI staff, the GenBank (KX284728.3) entry has now been updated with the corrected 16S and 23S sequences (Supplementary Table S1).

### Inclusion of Bacterial 16S Sequence in *Kappaphycus alvarezii* Chloroplast Assembly

**FIGURE 3:**
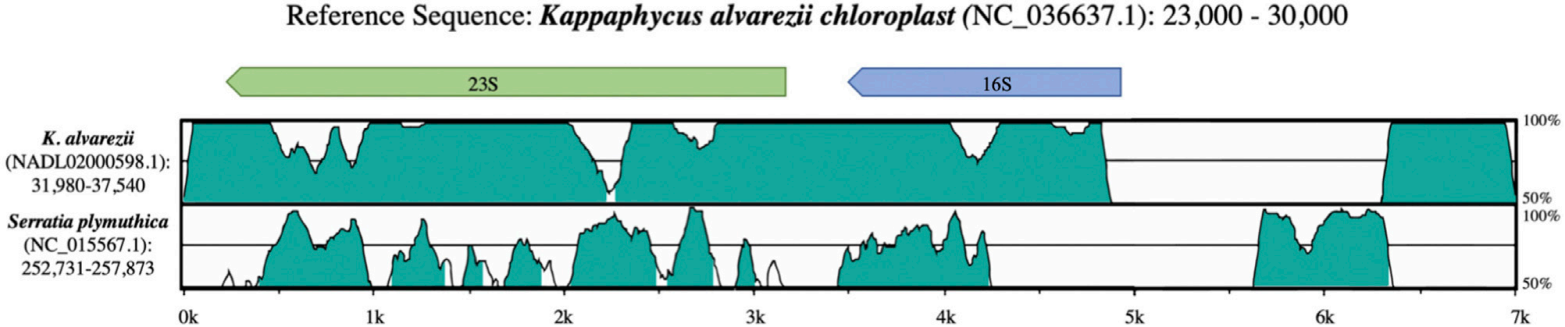
Alignment between rRNA sequences from the *K. alvarezii* chloroplast (reference), *K. alvarezii* whole genome assembly and *Serratia plymuthica*.

The *Kappaphycus alvarezii* chloroplast RefSeq genome (NC_036637.1) also appears to harbor contaminating sequences that do not belong as part of the reference. Alignments between this *K. alvarezii* chloroplast reference and assembled contigs generated from the original whole genome sequencing (WGS) project (NADL02000598.1) indicated that the *K. alvarezii* chloroplast reference harbors an additional 16S sequence (a ∼1.5 Kb insertion) immediately adjacent and upstream (NC_036637.1:27964-29353) of the annotated 16S sequence in the *K. alvarezii* chloroplast reference (Figure 3). This ∼1.5 kb insertion shares 88.40–88.52% identity with bacterial 16S sequences, with greatest similarity shared with *Serratia spp*. These findings strongly suggest that exogenous bacterial sequence was assembled as part of the chloroplast reference assembly. While there were also additional issues observed within the annotated 16S and 23S genes in the *K. alvarezii* chloroplast reference genome, the raw sequencing data used to generate these assemblies were unfortunately not available at the time of writing to further investigate this issue.

## Discussion

Our investigations into assembly errors detected in the above examined chloroplast genomes highlight the susceptibility of plastomes to erroneous bacterial sequence inclusion, specifically in regions of high sequence similarity, including the ribosomal RNA subunits. Due to the bacterial origin of chloroplasts, coupled with the evolutionary and functional constraints of the ribosomal RNA subunits, significant sequence conservation exists among the rRNA subunits of chloroplasts and bacteria. This sequence similarity can, as elucidated from the examples presented in this work, occasionally complicate the generation of an accurate chloroplast reference genome if bacterial reads are present and not removed prior to plastome assembly. While processes are in place to detect foreign sequence contamination during whole genome submissions, these processes are not run on chloroplast or other organelle sequences that may be submitted separately. Sequencing of pure plant samples can be very challenging, due to the presence of often diverse plant-associated microorganisms throughout the various tissues of the plant host. Indeed, the prevalence of associations between bacteria and plants leads to an almost inevitable bacterial presence in most plant DNA extracts (Rosenblueth et al., 2004).

In the examined chloroplast genomes, the bacterial sequences detected belonged to either previously described plant associated taxa, including close relatives of the plant hosts examined in this work, or to taxa that have been isolated from similar environmental niches. The published *Platanthera japonica* assemblies (NC_037440.1 and MN631092.1) examined in this work contain bacterial sequences most closely resembling *Klebsiella*. Members of the bacterial genus *Klebsiella* are capable of forming beneficial associations with orchids closely related to *P. japonica* (Pavlova et al., 2017) and *K. variicola* isolates can form endophytic relationships with plant hosts (Wei et al., 2014). Sequencing data and the resulting chloroplast assembly of the examined *Rhodochaete parvula* strain revealed the presence of bacterial sequences most closely resembling *Marinobacter*. Both *Marinobacter* and *R. parvula* inhabit similar niches in marine environments (Zuccarello et al., 2000; Rani et al., 2017). Sequences closely resembling *Serratia* were detected in the sequencing project and resulting chloroplast assembly from the red algae *Kappaphycus alvarezii*, and while this particular association has not been described, *Serratia* are known to associate with other plants (Asaf et al., 2017). Many of these potential bacterial-plant associations are one possible explanation for the presence of these reads in the examined sequencing data, however, bacterial sequences can also be introduced into samples through reagent and consumable contamination of extraction and library preparation kits (Salter et al., 2014). However, the amount of bacterial reads observed in the provided sequencing datasets was often quite substantial, often covering the main bacterial reference chromosome at >20X average fold coverage, a result which is inconsistent with passive or reagent contamination (Supplementary Table S1).

Regardless of the origin of these bacterial reads and sequences, our investigation indicates that the presence of these bacterial reads can lead to errors during the chloroplast assembly process. Due in part to the continuing decrease in sequencing costs, and similar to other sequence databases, plastid sequence databases are growing at an increasing pace. Our results suggest that additional quality screening is needed prior to plastid genome inclusion into these reference databases, with particular attention to regions coding for the ribosomal RNA genes. The assembly errors described in this work were all identified through comparisons with databases containing both bacterial and chloroplast sequences, and a similar approach is used by NCBI to identify suspicious contigs and sequences when uploading nuclear and other non-organelle specific genome assemblies. It is relevant to point out that the chloroplast assemblies investigated in this work were generated using a variety of methods and software (Supplementary Table S1). Furthermore, all but a single assembly were generated using a reference genome, suggesting that additional steps are needed to prevent the inclusion of contaminating bacterial sequences.

While this study is not exhaustive and there are likely to be many other types of errors in plastid reference genomes, given the biological samples and modern sample processing methods used to obtain plastid genomes, careful screening for bacterial data could provide some measure of confidence in the resulting genome. The use of tools designed to identify and exclude bacterial sequences from NGS datasets prior to plastid assembly, such as the ones employed in this work (see Methods), could help reduce or possibly eliminate similar errors in future plastid genome work. Failure to detect and correct these errors can lead to inaccurate predictions of presence/absence in metagenomic samples, improper representations of phylogenetic relationships (and ensuing inferences) and increases the probability of propagation of these errors through the use of incorrect references for assembly.

The chloroplast assemblies investigated in this work are representative of larger shortcomings in the field of genomics and highlight some of the vulnerabilities in publicly available sequence databases. None of these assemblies were previously flagged as potentially problematic, and even after we identified the suspicious nature and various inconsistencies in these assemblies, it was often difficult to track down the original data and submitters. Even when research groups reply to queries and share their raw sequencing data, which is not always the case, the investigation of such discrepancies coupled with further correspondence with busy researchers and the activation energy required to amend the database entries can substantially delay corrections to the public repositories. In some cases, additional sequencing data is required prior to submitting a correction, as was the case with the chloroplast entry for *Diplazium unilobum* (NC_035853.1), where we had contacted the original investigators and reported observing short stretches of bacterial-like sequences embedded within the annotated *D. unilobum* chloroplast 16S gene. After being provided the raw sequencing data (SRR16961501), our investigation clearly showed bacterial contamination caused the observed discrepancies. After rigorous treatment of the raw data for reads of bacterial origin, there remained insufficient chloroplast data to obtain a complete genome. Additional sequencing by the original authors was required prior to obtaining the correct sequence (SRR16974227), and this update has now been submitted during the completion of this manuscript (Supplementary Table S1). For bioinformaticists and comparative genomic researchers automating their data analyses using such reference genomes, an additional concern includes the fact that even once correct genomes are submitted to public repositories like GenBank to update those entries, such as with the *P. japonica* chloroplast genome (now MG925368.2), the RefSeq entry may not be updated, and in the case of *P. japonica* (NC_037440.1), this remained identical to the original erroneous submission until additional communication with NCBI staff resulted in a recent updated RefSeq record, over a year after the GenBank update (Supplementary Table S1). This work highlights the importance of being cautious when using public databases, as unintentional mistakes and errors are not always apparent nor easy to identify. Furthermore, the results presented here raise questions about the potential for similar issues to arise in plastomes of other organisms, particularly in bacterial-derived organelle genomes such as mitochondria. With these investigations, we also wish to emphasize the need to have access to original raw data to verify and validate original submissions and inconsistencies in assembled data, and to bring awareness of issues specific to the assembly of plastomes, with the goal of minimizing future mistakes and increasing overall genome data quality.

## Data Availability

The datasets presented in this study can be found in online repositories. The names of the repository/repositories and accession number(s) can be found in the article/Supplementary material.
